# Seven Letters with Answers

**Published:** 1875-01

**Authors:** 


### Our Correspondence.

### Letters Exposing Humbugs and Swindlers,

**With other Communications of Interest to the People.**

KINGSTON, ONT., Aug. 31, 1874.

DEAR SIR :—I noticed in a number, for 1873, I think, an item of news, that medical schools in Canada had at last received legal authority to claim and dissect certain bodies of deceased convicts, &c. Now the truth is that an act of Parliament passed in 1859, gives over for such purposes to the medical schools and medical practitioners having private pupils, unclaimed bodies of all convicts dying in prisons, hospitals and other institutions receiving government aid, and all coroners *are required*, before authorizing burial of bodies upon which they may have held inquest, the same being unclaimed, to notify the inspector of anatomy, that they may be made use of if required. You will thus see that we have long had the privilege.

O. Y., M. D.

The above from a prominent Canadian Physician, is self-explanatory.

---

JACKSONVILLE, FLA., Oct. 26, 1874.

DR. UP DE GRAFF :

We often see the BISTOURY here, and hear many favorable comments upon its usefulness. I came here last winter for my health, intended to have returned this summer, but was taken sick and could not get away. I now expect to stay during the coming winter unless my eyes get worse and compel me to come to your place for advice. Can you tell me what to do for my eyes ? I am aged 46. My vision has been very good until lately. In reading, the letters become blurred, the lines run together, and I find it very tiresome to read a page even, of a book. My eyes feel well enough, save when I try to read. They now become quite painful when I try to use them in that manner. I am wearing colored glasses and bathe them frequently during the day, but they grow no better. * * *

L.

Throw away your colored glasses, stop your "fussing" with your eyes, and have suitable spectacles fitted to them. That is all you need.

WARREN, O., Nov. 18, 1874.

DR. UP DE GRAFF :

A neighbor handed me a copy of the BISTOURY, in which I read an article upon "Spinal Curvature." Until I read this article I did not know what the trouble with our little child's back was. But I find upon examination that she has a crooked spine and that it is rapidly growing worse. She is six years old. Must I bring her to you to have a brace fitted, or can our family physician take the measurements for us, so that we can send to you for the instrument ? * * *

MRS. L. B.

It would be much the best plan to bring your child here for examination, so that we can decide *what sort* of a brace is needed. Unless your physician has had much experience with spinal difficulties, his judgment might not be the best with reference to the kind of instrument necessary to be used in the case.

---

NORFOLK, Va., Nov. 28, 1874.

DR. T. S. UP DE GRAFF :

We are advised to write you relative to the condition of our son, now 12 years of age. For two years he has been troubled with weak and inflamed eyes, until now he is almost blind, being unable to bear the light at all. He stays in a dark room, and says his eyes feel as though they were full of sand. I notice on each eye, just over the pupil, a white dot, the size of a pin's head, and when I separate the lids, the scalding tears run over his cheek. Now, what can we do for him ?

J. R. T.

Take him, without delay, to the nearest Oculist. He doubtless has a granular lid, with ulceration of the cornea. If the ulcers are allowed to spread, they will certainly ruin his eyes. Apply no patent "eye waters,"—they are usually composed of *acetate of lead*, which is fatal to ulcerated corneas.

---

SYRACUSE, N. Y., Dec. 1, 1874.

DR. UP DE GRAFF.

We saw Mrs.—— of Weedsport, upon whom you operated for *cataract* several years ago. She says that you informed her that the only way a cataract can be removed and the patient made to see, is by an operation. Dr.—— who comes to our place occasionally, says he can cure a cataract with medicine. He has been doctoring my mother for six months by rubbing stuff on her eye brows. It makes her see more light, but that is all. She don't seem to see any objects any better than she did before. Now what I want to know is, can he cure her with his medicine? If not, I want to send her to you.

J. S. T.

Traveling doctors resort to all manner of tricks to extort money from the afflicted. Trust them not. Cataract *cannot* be removed by medicines. By means of an almost painless operation, the "cataract" can be removed from her eye and her vision immediately restored.

---

WATSONTOWN, PA., Dec. 2, 1874.

DR. UP DE GRAFF.

We have a child six months old with what our physician calls, a double hare-lip. Is it old enough to be operated upon? He advises us to take it to you.

R.

Yes, it is old enough, but no harm will be done to wait longer if you choose. Any time, within a year old, will answer for an operation.

---

EUREKA, ALA., Nov., 25, 1874.

DR. UP DE GRAFF.

Our family physician directs us to write you relative to our infant, four months old. A few days after its birth, its eyes became inflamed, and in spite of all we could do, became worse and worse, until the lids were enormously swollen, and discharged great quantities of yellow matter. This continued for about three weeks, and then the swelling and inflammation subsided. When we were able to separate her eye-lids so as to look at her eyes, we found them somewhat sunken, and a white, or bluish looking "film" over them. We can not discover that she sees anything at all. What we want to know is, could those films be removed? If so, we will bring her to you at once, as we cannot bear to think of her always being blind. The Doctor gives us but little hope, but said we should write to you, asking your opinion, before we went so far to see you.

G. L. K.

The baby doubtless had *purulent ophthalmia,* a very dangerous form of disease of the eyes. The films you speak of, cannot be removed. Sorry to write it, but our opinion is, your baby is incurably blind.

